# Overexpression of TRIM24 Correlates with Tumor Progression in Non-Small Cell Lung Cancer

**DOI:** 10.1371/journal.pone.0037657

**Published:** 2012-05-30

**Authors:** Haiying Li, Liangliang Sun, Zhongping Tang, Lin Fu, Ying Xu, Zixuan Li, Wenting Luo, Xueshan Qiu, Enhua Wang

**Affiliations:** Department of Pathology, the First Affiliated Hospital of China Medical University and Department of Pathology, College of Basic Medical Sciences, China Medical University, Shenyang, Liaoning, China; Jawaharlal Nehru University, India

## Abstract

The objective of the current study was to investigate the expression pattern and clinicopathological significance of TRIM24 in patients with non-small cell lung cancer (NSCLC). The expression profile of TRIM24 in NSCLC tissues and adjacent noncancerous lung tissues was detected by immunohistochemistry. TRIM24 was found to be overexpressed in 81 of 113 (71.7%) human lung cancer samples and correlated with p-TNM stage (p  = 0.0006), poor differentiation (p = 0.004), Ki67 index (p<0.0001), cyclin D1(p = 0.0096) and p-Rb expression (p = 0.0318). In addition, depleting TRIM24 expression by small interfering RNA inhibited growth and invasion in lung cell lines. Moreover, TRIM24 depletion induced cell cycle arrest at the G1/S boundary and induced apoptosis. Western blotting analysis revealed that knockdown of TRIM24 decreased the protein levels of Cyclin A, Cyclin B, Cyclin D1, cyclin E and p-Rb and increased P27 expression. These results indicate that TRIM24 plays an important role in NSCLC progression.

## Introduction

Lung cancer is one of the leading causes of all cancer-related deaths worldwide and the incidence of lung cancer is increasing [1], [2]. Majority of the diagnosed lung cancer cases are non-small-cell lung cancers (NSCLCs). Although three therapeutic modalities (surgical resection, chemotherapy, and radiotherapy) have been established, long-term survival for lung cancer patients is still generally poor [3]. A variety of complex genetic, epigenetic, and microenvironmental factors play important roles in the survival and colonization of tumor cells at new locations [4], [5]. An improvement in the understanding of molecular processes involved in pulmonary carcinogenesis has led to new treatment options with targeted small molecules and vaccines demonstrating encouraging potential. Therefore, better defining the pathogenesis of lung cancer, looking for useful biomarkers, and exploring novel therapeutic targets are demanding tasks.

TRIM24 was originally named transcription intermediary factor 1-alpha (TIF1α), which was identified as a co-regulator of retinoid signaling [6]–[8]. Aberrant expression of TRIM24 might promote tumor development by multiple mechanisms. TRIM24 is a target of chromosomal translocations to form oncogenic fusion proteins in acute promyelocytic leukaemia, papillary thyroid carcinoma and myeloproliferative syndrome [9]–[11]. TRIM24 could ubiquitylate and negatively regulate p53 levels, which made TRIM24 a therapeutic target to restore tumor suppression by p53 [12]. TRIM24 also binds chromatin and oestrogen receptor to activate oestrogen -dependent genes which were associated with cellular proliferation and tumor development [13], [14]. Elevated expression of TRIM24 could promote progression of prostate cancer and negatively correlated with survival of breast cancer patients [14], [15]. These findings suggest that TRIM24 was an oncogene in tumor development. However, recent studies showed that loss of TRIM24 in mice led to hepatocellular carcinoma development and TRIM24 interacted with TRIM28 and TRIM33 to form regulatory complexes that suppressed murine hepatocellular carcinoma, suggesting its role as a tumor suppressor in heptocellular carcinoma [16]. In addition, arterial calcifications and expression of vitamin D receptor targets were increased in mice lacking TRIM24, showing that TRIM24 could prevent calcification of arteries by decreasing the activity of the vitamin D signaling pathway [17].

The protein expression of TRIM24 in primary lung cancer and its relationship with clinicopathological factors have not yet been examined. In addition, the biological roles of TRIM24 in lung cancer cells are still unclear. In order to address the above questions, we examined TRIM24 expression in non-small-cell lung cancer tissues by immunohistochemistry. In addition, we also explored the association of TRIM24 with proliferation and invasion ability in several lung cancer cell lines.

## Results

### Overexpression of TRIM24 Protein in Non-small Cell Lung Cancer Tissues

**Figure 1:**
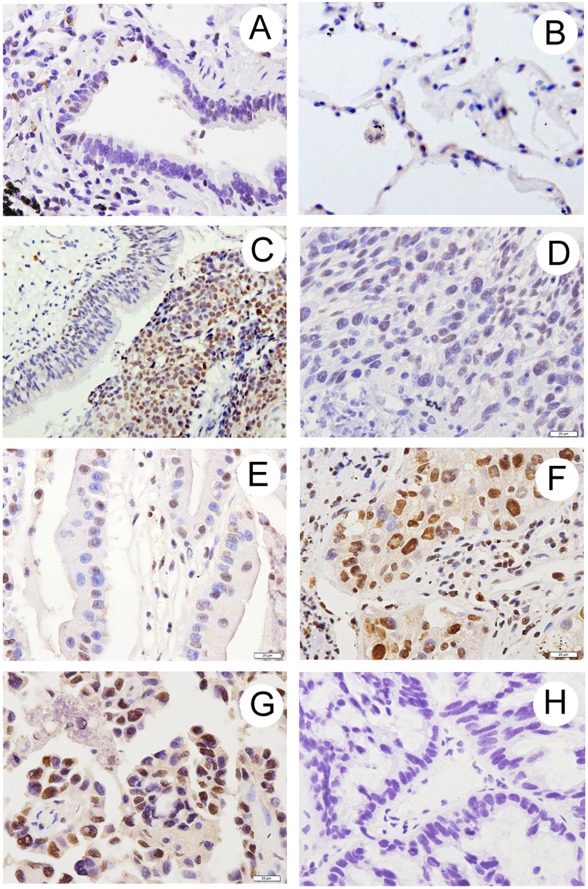
Immunohistochemical staining of TRIM24 in lung cancer tissue sections. A. Negative staining in normal bronchial epithelium in non-cancerous lung tissue. B. Negative staining in normal pneumocytes in the alveoli of non-cancerous lung tissue. C. TRIM24 immunostaining was negative in bronchial epithelium adjacent to lung adenocarcinoma. D. Negative TRIM24 staining in a case of Stagel, moderately differentiated squamous cell carcinoma. E. Negative staining in Stagel, well differentiated lung adenocarcinoma. F. Positive TRIM24 staining in a case of Stage II, poorly differentiated squamous cell carcinoma. G. Positive TRIM24 staining in a case of Stage III, moderately differentiated adenocarcinoma. H. Negative control using rabbit immunoglobulin.

**Figure 2:**
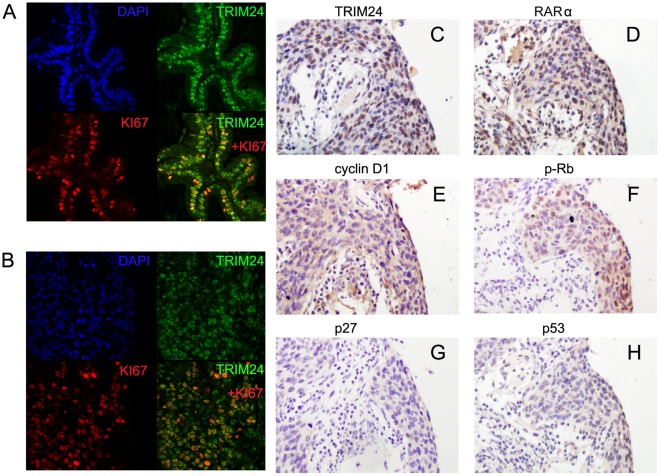
TRIM24 expression in NSCLC tissues was associated positively with the expression of Ki-67, cyclinD1 and p-Rb. Immunofluorescence staining showed co-localization of TRIM24 and Ki-67 in adenocarcinoma (A) and squamous cell carcinoma (B). Immunohistochemical staining for TRIM24 (C) and RARα (D), cyclinD1 (E), p-Rb (F), p27 (G) and p53 (H) staining in a case of NSCLC.

We analyzed the expression of TRIM24 in 113 NSCLC specimens and their corresponding normal tissues by immunohistochemistry. TRIM24 expression was observed in nuclear compartments of tumor cell (Figure 1 C–G), while the normal bronchial epithelia and pneumocytes exhibited negative or low staining (Figure 1 A, B). The staining intensity of normal respiratory epithelium adjacent to tumor could be evaluated in several sections containing malignant tumors and normal tissues in the same slide. Whereas none to weak staining for TRIM24 was detected in the normal lung tissues, a strong staining of TRIM24 was detected in adjacent tumor cells (Figure 1 C). We investigated the relationship between the total TRIM24 expression and the clinical parameters. As shown in Table 1, no statistical difference was found between the TRIM24 overexpression and the characteristics of age (p = 0.4697), gender (p = 0.1814), tumor status (p = 0.1812), nodal status (p = 0.0825) and tumor type (p  = 0.6327). However, patients with high TRIM24 expression showed poor differentiation (p = 0.004) and had advanced stage of NSCLC (I vs II + III + IV, p = 0.0006). We examined the expression of Ki-67 to investigate the relationship between TRIM24 positivity and proliferative activity of cancer tissues by immunohistochemistry. Cases that had high levels of TRIM24 expression tended to have high proliferation index indicated by Ki-67 labeling (p<0.0001). Double immunofluorescence analysis was performed and co-localization of Ki-67 and TRIM24 was observed (Figure 2A, B). Immunostaining for p53, retinoic acid receptor alpha (RARα), cyclin D1, p-Rb, and p27 were also performed and their association with TRIM24 expression was analyzed (Figure 2 C–H). As shown in Table 1, TRIM24 overexpression correlated with high cyclin D1 (p = 0.0096) and p-Rb expression (p = 0.0318), while no statistical difference was found between the TRIM24 overexpression and p53 (p = 0.9028), RARα (p = 0.1025), and p27 status (p = 0.6335).

**Table 1:** Distribution of TRIM24 status in NSCLC according to clinicopathological characteristics.

Characteristics	Number of patients	TRIM24 negative	TRIM24 positive	*P*
Age				
<60	61	19(31.15%)	42(68.85%)	0.4697
≥60	52	13(25.00%)	39(75.00%)	
Gender				
Male	63	21(33.33%)	42(66.67%)	0.1841
Female	50	11(22.00%)	39(78.00%)	
Histology				
Adenocarcinoma	71	19(26.76%)	52(73.24%)	0.6327
Squamous cell carcinoma	42	13(30.95%)	29(69.05%)	
Differentiation				
Well	50	21(42.00%)	29(58.00%)	0.0040
Moderate- Poor	63	11(17.46%)	52(82.54%)	
TNM stage				
I	49	22(44.90%)	27(55.10%)	0.0006
II+III+IV	64	10(15.63%)	54(84.38%)	
Tumor status				
T1	39	8(20.51%)	31(79.49%)	0.1812
T2 T3 T4	74	24(32.43%)	50(76.57%)	
Nodal status				
N0	67	23(34.33%)	44(65.67%)	0.0825
N1 N2 N3	46	9(19.57%)	37(80.43%)	
Ki67				
Low	56	25(44.64%)	31(55.36%)	0.0001
High	57	7(12.28%)	50(87.72%)	
RARα				
Low	64	22(34.38%)	42(65.63%)	0.1025
High	49	10(20.41%)	39(79.59%)	
Cyclin D1				
Low	63	24(38.10%)	39(69.10%)	0.0096
High	50	8(16.00%)	42(84.00%)	
p-Rb				
Low	56	21(37.50%)	35(62.50%)	0.0318
High	57	11(19.30%)	46(80.70%)	
p27				
Negative	57	15(26.32%)	42(73.68%)	0.6335
Positive	56	17(30.36%)	39(69.64%)	
p53				
Low	59	17(28.81%)	24(71.19%)	0.9028
High	54	15(27.78%)	39(72.22%)	

### TRIM24 Depletion Inhibits Proliferation, Invasion and Induces Apoptosis in Lung Cancer Cell Lines

**Figure 3:**
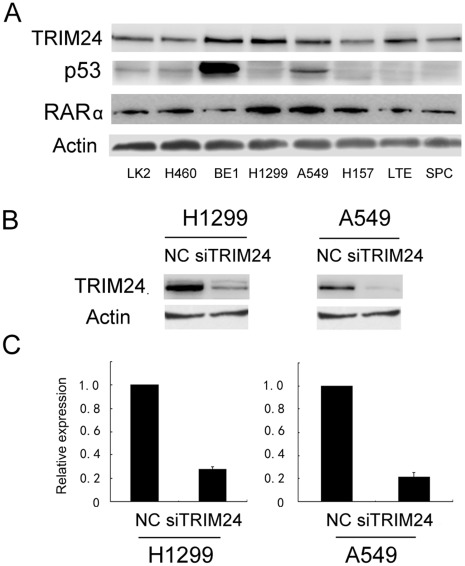
TRIM24 depletion in A549 and H1299 cell lines. A. Expression levels of TRIM24, p53 and RARα were analyzed by western blot in a panel of lung cancer cell lines. B. Western blot of TRIM24 depletion efficiency in cancer cells. C. Real-time PCR analyses of TRIM24 depletion efficiency in cancer cells.

**Figure 4:**
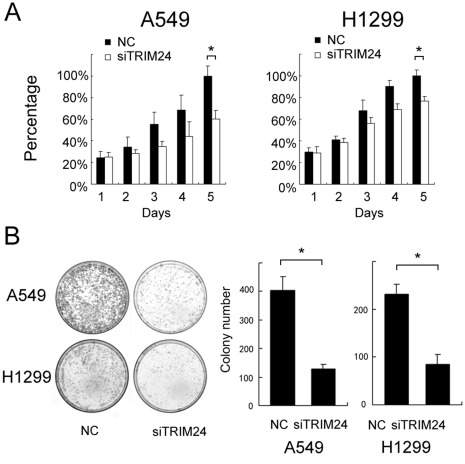
TRIM24 depletion impaired cancer cell proliferation. A. MTT assay was performed after TRIM24 siRNA treatment. A reduction of absorbance was observed (p<0.05 at day 5 for both A549 and H1299). B. Assessment of clonogenic potentials of the TRIM24-depleted cancer cells. Numbers of colonies were counted. The number of colonies formed by cells treated with TRIM24 siRNA was far less than that of control cells (p<0.05). Columns, mean; bars, SD. *p<0.05.

**Figure 5:**
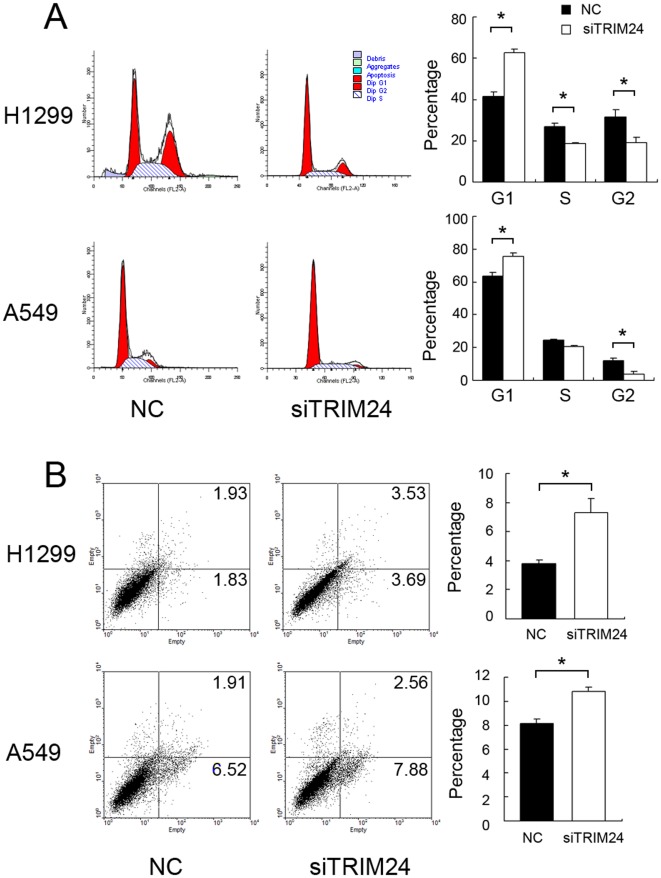
TRIM24 knockdown inhibited cell cycle progression and induced apoptosis. The percentage of G1 phase was increased in cells with TRIM24 knockdown (H1299 and A549, p<0.05), whereas the percentages of S phase (H1299, p<0.05) and G2 phase (H1299 and A549, p<0.05) were decreased in these cells compared with control cells (A). TRIM24 knockdown also induced cell apoptosis in both A549 and H1299 cell lines (B, p<0.05). *p<0.05.

Expression of TRIM24 was analyzed by western blot in a panel of lung cancer cell lines (Figure 3). We found the level of TRIM24 expression in H1299 and A549 cells was higher than other cell lines. Since the role of TRIM24 is closely associated with p53 and RARα in some types of tumor, we also examined p53 expression and RARα in lung cancer cell lines. There was no obvious association between these factors. In order to explore the biological function of TRIM24 in lung cancer, we employed siRNA to knockdown TRIM24 expression in both H1299 and A549 cell lines. TRIM24-specific siRNA considerably reduced both mRNA as well as protein expression levels of TRIM24 after 48 hours of siRNA treatment (Figure 3). Our cell proliferation analysis showed that the depletion of TRIM24 in H1299 and A549 cells led to a significant reduction of the proliferation rate (A549 39% reduction at day 5, p<0.05; H1299 23% reduction at day 5, p<0.05) and foci numbers as well as sizes (A549 control vs TRIM24si: 400±38 vs 130±20, p<0.05; H1299 control vs TRIM24si: 235±25 vs 85±18, p<0.05), suggesting that TRIM24 modulates proliferation of lung cancer cells (Figure 4). Analysis of cell cycle showed that in TRIM24 knockdown cells the percentage of S and G2 phase was much lower than control cells and the percentage of G1 phase was increased (Figure 5A). Thus TRIM24 knockdown inhibited cell cycle progression.

In addition, Annexin V kit was employed to characterize the death feature of H1299 and A549 cells with TRIM24 knockdown (Figure 5B). Clearly, a significant population of early and late apoptosis (H1299: 7.22%; A549: 10.45%) was observed in cells with TRIM24 knockdown compared with scramble controls (H1299: 3.76%; A549: 8.43%), demonstrating that TRIM24 knockdown results in apoptosis of the lung cancer cells, especially in H1299 cells which have high endogenous TRIM24 expression.

**Figure 6:**
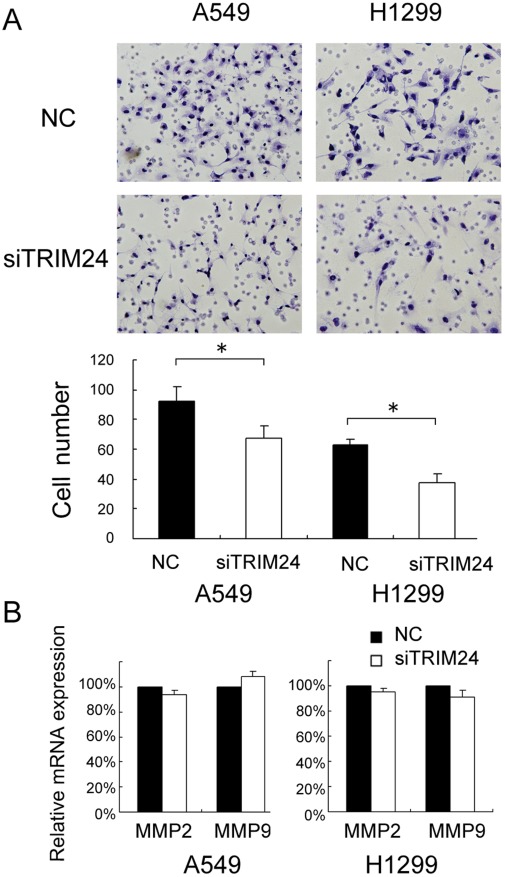
Invasion assays of A549 and H1299 cells transfected with control and TRIM24-specific siRNA. TRIM24 siRNA treatment have a measurable blocking effect on cell invasion in both cell lines. Numbers of cells invading onto the lower surface of the filter were counted, a significant difference was observed (A, p<0.05). Columns, mean; bars, SD. There was no significant change of MMP2 and MMP9 expression levels after TRIM24 knockdown (B). *p<0.05.

To determine whether TRIM24 contributes to the invasion of non-small cell lung cancer cells, we conducted matrigel invasion assays. As shown in Figure 6A, TRIM24 knockdown inhibited cell invasion (A549 control vs TRIM24si: 91±15 vs 68±11, p<0.05; H1299 control vs TRIM24si: 62±4 vs 38±8, p<0.05).

To further explore the mechanisms by which TRIM24 promotes NSCLC cell invasion, we explored the expression of MMP-2 and MMP-9 expression before and after transfection of siRNA. As shown in Figure 6B, the mRNA levels of MMP-2, MMP-9 were examined by Quantitative Real-time RT-PCR. However, we did not observe remarkable changes of their expression.

### Depletion of TRIM24 Downregulated CyclinA, B, D1 and E Expression and Upregulated P27 in Lung Cancer Cells

**Figure 7:**
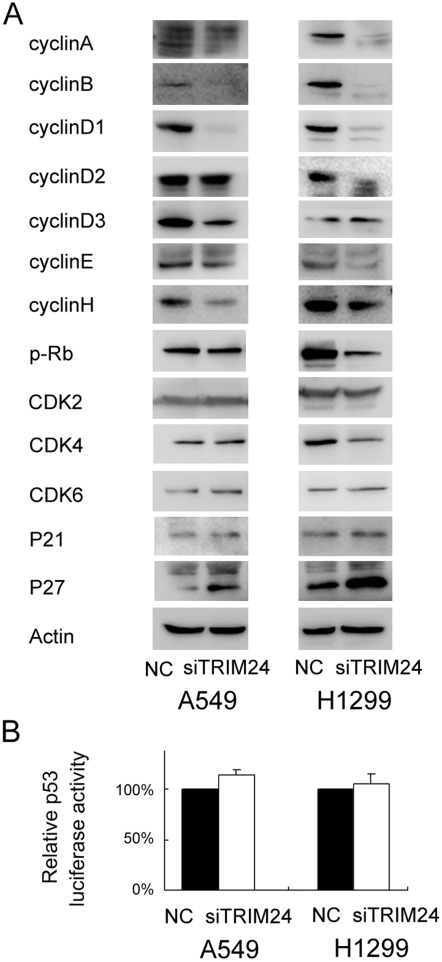
Expression of cell-cycle related molecules levels and p53 luciferase activity in TRIM24 depleted H1299 and A549 cells. Western blot analysis of a series of cell cycle related factors showed the protein levels of Cyclin A, B, D1, E and p-Rb were decreased and P27 expression was increased after silencing TRIM24 in H1299 and A549 cells (A). There was no significant change of p53 luciferase activity after siRNA treatment in both cell lines (B).

The cell cycle analyses were performed in cancer cells with or without TRIM24 knockdown, and found that the percentage of G1 phase was increased in cells with TRIM24 knockdown, whereas the percentage of S phase was decreased in these cells compared with control cells (Figure 5). These results indicate that TRIM24 depletion induces cell cycle arrest at the G1/S boundary. Also, the proportion of G2 phase cells was decreased. To investigate the mechanism underlying cell cycle arrest, we tested the effect of TRIM24 knockdown on Cyclin A, Cyclin B, Cyclin D1, Cyclin D3, Cyclin E, CDK2, CDK4, CDK6, P-Rb, P21 and P27 levels. As shown in Figure 7A, Western blotting analysis revealed that knockdown of TRIM24 decreased the protein levels of Cyclin A, B, D1, E, p-Rb and increased P27 expression. Together, these results suggest that inhibiting TRIM24 expression induces cell cycle arrest at the G1-S transition and suppresses lung cancer cell growth. To directly measure p53 transcriptional activity, a p53-responsive luciferase reporter plasmid was used to examine the relationship between TRIM24 and p53 activity in lung cancer cell lines. There was no significant change of cellular p53 activity after TRIM24 knockdown (Figure 7B).

## Discussion

Up-regulation of TRIM24 expression had been implicated in several human cancers such as acute promyelocytic leukaemia, papillary thyroid carcinoma and breast cancer [9]–[11], [14]. Moreover, TRIM24 overexpression correlated with survival of breast cancer patients [14], [15]. However, the expression pattern of TRIM24 as well as its correlation with clinical and pathological factors has not yet been defined in human lung cancer. In this study, we demonstrated that TRIM24 protein expression in lung cancer tissues was higher than that in corresponding normal lung tissues. There was a close correlation between TRIM24 up-regulation and pTNM stage and differentiation.

Previous research concerning its expression pattern showed that TRIM24 was overexpressed in breast cancers at both mRNA and protein levels and its overexpression was correlated with ER, PR status and poor prognosis [15]. Our study found a correlation between TRIM24 overexpression and pTNM stage and poor differentiation in lung cancer, which was in accord with previous data, suggesting TRIM24 may play an important role in lung cancer progression. To validate the potential role of TRIM24 in lung cancer development, we first checked its expression level in several cell lines and picked up A549 and H1299 with relatively high TRIM24 level for further study. We employed siRNA to knockdown TRIM24 expression in these two cell lines. We found an impaired proliferation capacity and colony formation ability of both A549 and H1299 cells after TRIM24 knockdown. Furthermore, matrigel invasion assay showed decreased invading ability of siRNA treated cells. Thus, our study suggested that TRIM24 functioned as an oncogene in lung cancer development.

Previous report indicated that TRIM24 can directly ubiquitinate p53 and negatively regulate protein level of P53 in breast cancer cell lines, implying its roles in proliferation and apoptosis [12]. Most of the proliferative factors influence cell growth by affecting cell cycle progression. So we employed cell cycle analysis and found that TRIM24 knockdown cells showed higher levels of G1 phase and lower S phase than the control cells. So TRIM24 knockdown inhibited G1 to S transition in cell cycle progression, which might explain the mechanism of TRIM24 on lung cancer cell proliferation. TRIM24 knockdown also induced apoptosis in H1299 and A549 cells, which may partially due to blockage of G1/S transition. In addition, we showed that TRIM24 siRNA blocked cell invasion. To further explore the mechanism, we explored the expression of invasion-related MMP2 and MMP9. We did not observe remarkable changes to these molecules. It could be argued that there are other functional aspects of TRIM24 contributing to the regulation, which needs further investigation.

To find out the potential mechanism of TRIM24 on cell cycle regulation, we examined the effect of TRIM24 knockdown on a number of cell-cycle related molecules. We checked the expression of cyclinA, B, D1, D2, D3, E, H, CDK2/4/6, p-Rb, p21 and P27. We found that the levels of cyclinA, B, D1, E and p-Rb were decreased after TRIM24 knockdown, while the level of P27 expression was elevated. Cyclin D1 interacts with Cdk4/6 to form a complex phosphorylating Rb, which regulates cell proliferation by controlling progression through the restriction point within the G1-phase of the cell cycle [18]. Cyclin D1 was overexpressed in a variety of cancers and associated with cancer cell proliferation [19]–[21]. Cyclin A is required for cells to progress through the S phase [22]. Cyclin B is a mitotic cyclin and its accumulation is only found at the G2-M transition [23]. The tumor suppressor protein p27 acts as an inhibitor of cell cycle progression. In association with CDK2 complexes, P27 serves to inhibit kinase activity and block progression through G1/S [24], [25]. Thus our results showing decreased level of cyclin A, B, D1, E, p-Rb and increased p27 protein correlated with the fact of decreased level of S and G2 phase cells and increased G1 phase cells after TRIM24 knockdown, suggesting TRIM24 plays an important role in cell cycle control of lung cancer cells. Furthermore, TRIM24 overexpression was associated with high levels of cyclin D1 and p-Rb in lung cancer specimens.

Some data showed that the role of TRIM24 was associated with p53 and retinoic acid receptor alpha. We examined these two factors in clinical samples and cell lines. However, we did not find significant relationship between TRIM24 and these factors. Furthermore, no obvious change of cellular p53 activity was observed after TRIM24 knockdown in A549 (wild-type p53) and H1299 (p53 null) cell lines using luciferase reporter system, suggesting the role of TRIM24 on cell cycle regulation was independent of p53 activity.

In conclusion, the present study investigated the expression pattern and clinicopathological significance of TRIM24 in NSCLC and addressed the biological role and potential mechanism of TRIM24 in lung cancer progression.

## Materials and Methods

### Patients and Specimens

This study was conducted with the approval of the local institutional review board at the China Medical University. 113 cases of NSCLC samples were obtained from the First Affiliated Hospital of China Medical University during the period of 2007 to 2009. The histological diagnosis and grade of differentiation of the tumors were defined by evaluation of the hematoxylin and eosin-stained tissue sections, according to the World Health Organization guidelines of classification. All 113 specimens were re-evaluated with respect to their histological subtypes, differentiation status, and tumor stages. For NSCLC samples, Squamous cell carcinoma and adenocarcinoma were identified in 42 and 71 of the 113 cases, respectively. Lymph node metastases were observed in 46 patients. The p-TNM taging system of the International Union Against Cancer (7th Edition) was used to classify specimens as stages I (n = 49), II (n = 35), III and IV (n = 29).

### Cell Lines

NHBE, A549, H1299, H157, H460, and H1299 cell lines were obtained from American Type Culture Collection (Manassas, VA, USA). SPC, LTE, and LK2 cell lines were purchased from Shanghai Cell Bank of Chinese Academy of Science. BE1 cell line was a gift from Dr. J Zheng (Department of Pathology, Peking University, Beijing). The cells were cultured in RPMI 1640 (Invitrogen, Carlsbad, CA, USA) containing 10% fetal calf serum (Invitrogen), 100 IU/ml penicillin (Sigma, St. Louis, MO, USA), and 100 µg/ml streptomycin (Sigma). Cells were grown on sterile tissue culture dishes and were passaged every 2 days using 0.25% trypsin (Invitrogen).

### Immunohistochemistry

Surgically excised tumor specimens were fixed with 10% neutral formalin, embedded in paraffin and 4 µm thick sections were prepared. Immunostaining was performed using the avidin–biotin–peroxidase complex method (Ultra Sensitive TM, Maixin, Fuzhou, China). The sections were deparaffinized in xylene, rehydrated in graded alcohol series and boiled in 0.01 M citrate buffer (pH 6.0) for 2 minutes in an autoclave. Endogenous peroxidase activity was blocked using hydrogen peroxide (0.3%), which was followed by incubation with normal goat serum to reduce non-specific binding. Tissue sections were incubated with TRIM-24 rabbit polyclonal antibody (1∶150 dilution) (Proteintech, Chicago, IL, USA). Rabbit immunoglobulin was used as a negative control. Immunohistochemical stainings for Ki67 (1∶200 dilution) (Maixin, Fuzhou, China), RARα (1∶200 dilution) (Santa Cruz Biotechnology, Santa Cruz, CA, USA), p53 (1∶200 dilution) (DO-7, Santa Cruz Biotechnology), cyclin D1 (1∶100 dilution) (Cell signaling technology, Boston, MA, USA), p-Rb (1∶200 dilution) (Cell signaling technology, Boston, MA, USA) and p27 (1∶150 dilution) (Santa Cruz Biotechnology) were also performed. Staining for all primary antibodies was performed at room temperature for 2 hours. Biotinylated goat anti-mouse serum IgG, Biotinylated goat anti- rabbit serum IgG(ready-to-use ) (Maixin, Fuzhou, China) was used as the secondary antibody. After washing, the sections were incubated with horseradish peroxidase-conjugated streptavidin–biotin, followed by 3, 3′-diaminobenzidine tetrahydrochloride to develop the peroxidase reaction. Counterstaining of the sections was done with hematoxylin, which were then dehydrated in ethanol before mounting.

Two independent investigators examined all tumor slides randomly. Five views were examined per slide, and 100 cells were observed per view at 400×magnification. Immunostaining of TRIM24 was scored following a semi-quantitative scale by evaluating in representative tumor areas, the intensity and percentage of cells showing higher immunostaining than the control cells. nuclear staining of the tumor cells was considered as positive immunostaining. The intensity of TRIM24 nuclear staining was also scored as 0 (no staining), 1 (weak), 2 (marked). Percentage scores were assigned as 1- 1–25%, 2- 26–50%, 3- 51–75% and 4- 76–100%. The scores of each tumor sample were multiplied to give a final score of 0 to 8 and the total expression of TRIM24 was determined as either negative or low expression (−): score <4 or overexpression (+): score ≥4. The immunohistochemical staining for Ki-67 was evaluated and scored as the percentage of cancer cells with nuclear immunoreactivity with a total of 500 tumor cells examined per slide. The median value of this series (35% of positive cells) was used as a threshold value to distinguish tumors with low (<35%) versus high (≥35%) index of cell proliferation. According to previous criteria for evaluating cyclin D1, p53, pRb expression, we determined their level as high or low/negative expression [26]. p27 expression was determined as positive or negative according to previous report [27]. According to previous criteria, RARα expression was determined as high expression or low expression [28].

### Immunofluorescence

4-µm sections were deparaffinized in xylene, rehydrated in graded alcohol series and boiled in 0.01 M citrate buffer (pH 6.0) for 2 minutes in an autoclave. Double immunofluorescence analysis was performed using mouse monoclonal antibody to Ki67 (Maixin), and rabbit polyclonal antibody to TRIM24. Goat anti-rabbit (Alexa Fluor 488 labeled; Molecular Probes) and goat anti-mouse (Alexa Fluor 594 labeled; Molecular Probes) were used as secondary antibodies. Fluorescence signals were analyzed by recording stained images using Olympus FV1000 Laser Scanning Confocal Microscope.

### Quantitative Real-time PCR (SYBR Green Method)

Quantitative real-time PCR was performed using SYBR Green PCR master mix (Applied Biosystems) in a total volume of 20 µl on 7900HT Fast Real-Time PCR System (Applied Biosystems) as follows: 95°C for 30 seconds, 40 cycles of 95°C for 5 seconds, 60°C for 30 seconds. A dissociation step was performed to generate a melting curve to confirm the specificity of the amplification. β-actin was used as the reference gene. The relative levels of gene expression were represented as ΔCt = Ct gene-Ct reference, and the fold change of gene expression was calculated by the 2-ΔΔCt method. Experiments were repeated in triplicate. The primer sequences are as followers: TRIM24 forward, 5′ CGCCACCCAAGTTGGAGT 3′, TRIM24 reverse, 5′ GCTGGGAACCTCAGTAGTGTCCT 3′; β-actin forward, 5′ ATAGCACAGCCTGGATAGCAACGTAC 3′, β-actin reverse, 5′ CACCTTCTACAATGAGCTGCGTGTG 3′. MMP2 forward, 5′-TGTGTTCTTTGCAGGGAATGAAT-3′; MMP2 reverse, 5′-TGTCTTCTTGTTTTTGCTCCAGTTA-3′; MMP9 forward, 5′-CCTCTGGAGGTTCGACGTGA-3′; MMP9 reverse, 5′-TAGGCTTTCTCTCGGTACTGGAA-3′;

### Western Blot Analysis

Total proteins from cell lines were extracted in lysis buffer (Thermo Fisher Scientific,Rockford,IL) and quantified using the Bradford method. Fifty micrograms of protein were separated by SDS–PAGE (12%). After transferring, the polyvinylidene fluoride (PVDF) membranes (Millipore, Billerica, MA, USA) were incubated overnight at 4°C with the following antibodies -TRIM24(1∶1000; Proteintech, Chicago, IL, USA), p53 (DO-7, 1∶500), RARα (1∶500), beta-Actin(1∶500; Santa Cruz Biotechnology, Santa Cruz, CA), cyclin A(1∶1000), cyclin B(1∶1000), cyclin D1(1∶1000), cyclin D2(1∶1000), cyclin D3(1∶1000), cyclin E(1∶800), CDK2(1∶1000), CDK4(1∶1000), CDK6(1∶1000), p-Rb(1∶2000), P21(1∶800), P27(1∶800) (Cell signaling technology, Boston, MA, USA). After incubation with peroxidase-coupled anti-mouse/rabbit IgG (Santa Cruz Biotechnology) at 37°C for 2 hours, bound proteins were visualized using ECL (Thermo Fisher Scientific) and detected using BioImaging Systems (UVP Inc., Upland, CA, USA). The relative protein levels were calculated based on β-actin as the loading control.

### Small Interfering RNA Treatment

On-TargetPlus SMARTpool siRNA for TRIM24 (M-005387-03-0005) and ON-TARGETplus Non-targeting siRNA #1 (D-001810-01-20) were purchased from Dharmacon. For transfections, cells were seeded in a 24-well plate 24 h before the experiment. The cells were transfected with siRNA using the DharmaFECT 1 (0.20 µL/well; ThermoFisher Scientific) according to the manufacturer’s protocol. Following transfection, the mRNA and protein levels were assessed 48 hours later.

### Cell Proliferation Test and Colony Formation Assay

Cell proliferation assay was performed using Cell Counting Kit-8 solution (Dojindo, Gaithersburg, MD) according to the manufacturer’s protocol. Briefly, cells were seeded at a concentration of 5×10^3^ cells/100 µl/well in 96-well culture plates and treated with 10 µl/well of Cell Counting Kit-8 solution during the last 4 hours of the culture. Optical density of the wells was measured at 450 nm using a microplate reader. For colony formation assay, cells were planted into three 6-cm cell culture dishes (1000 per dish for A549 and H1299 cell lines) and incubated for 12 days. Plates were washed with PBS and stained with Giemsa. The number of colonies with more than 50 cells was counted.

### Cell Cycle Analysis

Cells(500,000) were seeded into 6-cm tissue culture dishes. Twelve hours later, cells were transfected with indicated amounts of siRNA. Cells were synchronized after a 20 h serum starvation and then time points taken at 24 h after application of 10% serum media to gauge effects on the cell cycle. Cells were harvested, fixed in 1% paraformaldehyde, washed with phosphate-buffered saline (PBS) and stained in 5 mg/ml propidium iodide in PBS supplemented with RNase A (Roche, Indianapolis, IN) for 30 minutes at room temperature. Data were collected using BD systems.

### Matrigel Invasion Assay

Cell invasion assay was performed using a 24-well Transwell chamber with a pore size of 8 µm (Costar,Cambridge,MA). The inserts were coated with 20 µl Matrigel (1∶3 dilution, BD Bioscience, San Jose, CA, USA). Forty-eight hours after the transfection, cells were trypsinized and 3×10^5^ cells in 100 µl of serum-free medium were transferred to the upper Matrigel chamber and incubated for 16 hours. Medium supplemented with 10% FBS alone or containing 100 ng/ml EGF (Invitrogen,Carlsbad,Carlsbad,CA) was added to the lower chamber as the chemoattractant. After incubation, the non-invaded cells on the upper membrane surface were removed with a cotton tip, and the cells that passed through the filter were fixed with 4% paraformaldehyde and stained with hematoxylin. The number of invaded cells was counted in 10 randomly selected high power fields under the microscope. This experiment was performed in triplicate.

### Apoptosis Analysis

For detection of apoptosis, adherent cells were both collected and resuspended in cold PBS for analysis. Cells were stained with Annexin V-FITC Apoptosis Kit (BD Pharmingen, USA) to monitor apoptosis cells and propidium iodide (PI) to detect dead cells. Data were collected using BD systems.

### Transient Transfection and Luciferase Reporter Assay

Reporter gene transfection and luciferase activity assay cells in 80 confluent growing on a 24 well plates were co-transfected with the firefly luciferase reporter of P53 containing a TA promoter (pp53-TA-luc, Beyotime Biotechnology, China) (0.2 µg) along with the Renilla luciferase reporter (Promega Co.) (0.02 µg) for 12 h using a attractene reagent (QIAGEN) according to the protocols supplied by manufacturers.

The luciferase activity was measured in the cellular extracts using a dual luciferase reported gene assay kit (Promega, CA, USA). The relative activity of reporter gene was calculated by dividing signals of p53 luciferase reporter by signals obtained form Renilla luciferase reporter.

### Statistical Analysis

SPSS version 16.0 for Windows was used for all analyses. The Chi-squared test was used to examine possible correlations between TRIM24 expression and clinicopathologic factors. The Student’s t-test was used to compare other data. p value was based on the two-sided statistical analysis, and p<0.05 was considered to indicate statistical significance.
